# Laser-guided transtibial technique improved single-bundle reconstruction of anterior cruciate ligament

**DOI:** 10.1186/s13018-018-0878-y

**Published:** 2018-07-25

**Authors:** Zhen Yuan, Ning Bian, Yuefeng Hao, Lu-jie Zong, Yu Kou, Dan Hu

**Affiliations:** 1 Center of Sports Medicine and Rehabilitation, The Affiliated Suzhou Hospital of Nanjing Medical University, 242 Guangji Road, Suzhou, Jiangsu 215008 People’s Republic of China; 2 Medical College of Soochow University, No. 199 Renai Road, Suzhou, Jiangsu 215000 People’s Republic of China

**Keywords:** Anterior cruciate ligament, Transtibial tunnel technique, Anatomical reconstruction, Double-sided laser technology

## Abstract

**Background:**

The transtibial tunnel technique achieves equal length reconstruction of the anterior cruciate ligament (ACL). This study aimed to investigate whether transtibial tunnel technique can achieve anatomical reconstruction of ACL.

**Methods:**

For 25 corpses, the anterior soft tissue of the knee joint was detached so that the ligamentous surface was fully exposed, then the knee joint was fixed at 90° with an external fixator and the anterior cruciate ligament was removed. Double-sided laser technology was used to establish spatial conformation of ACL.

**Results:**

The male to female ratio of the subjects was 19:6, with an average age of 59.52 ± 11.13 years. Patellar tendon length was 35.23 ± 5.10 mm, tibial eminence length and width was 15.75 ± 2.44 and 7.80 ± 1.28 mm, respectively, and femoral attachment length and width was 15.40 ± 2.17 and 8.97 ± 1.61 mm, respectively. When the flexion turned 90°, the tibial tunnel length was 31.83 ± 4.09 mm and the distance to the tibial plateau, patellar tendon, and medial collateral ligament was 16.33 ± 4.56, 10.79 ± 5.85, and 23.12 ± 5.99 mm, respectively.

**Conclusions:**

With the aid of double-sided laser technology, transtibial tunnel technique can safely achieve single-bundle reconstruction of ACL.

## Background

The injury of anterior cruciate ligament (ACL) has high incidence in sports medicine. The annual incidence of ACL is about 1‰ in general population in Sweden, and the average age is only 32 years old [1]. In China, the incidence of ACL injury has increased recently. Long-term conservative treatment will result in unstable joints, injury of attached structures, and osteoarthritis [2]. Therefore, surgery is usually recommended, and ACL reconstruction based on transtibial tunnel technique is the most common surgical method [3, 4]. However, the femoral tunnel is relatively high, and the graft is too vertical to control the rotation, which will result in the injury of the meniscus and osteoarthritis [5, 6]. Anatomical reconstruction may solve this problem by reconstructing the natural ACL spatial structure analogy [7].

Several studies have shown that anatomical structure reconstruction, whether single bundle or double bundles, could effectively improve the stability of joints, increase the recovery rate and sustain the time, and prevent abnormal rotation and joint laxity [8, 9]. Therefore, anatomical reconstruction becomes a new trend of ACL reconstruction [2]. Currently, there are two main techniques for anatomical reconstruction: trans-portal (TP) and outside-in (OI) [10, 11]. Different techniques have different advantages and disadvantages [12, 13]. Traditional transtibial tunnel (TT) technique has advantages such as fewer incisions and ease to place graft, but it is still questioned because of its non-anatomical position [6]. Current literatures support that it is impossible to conduct anatomical reconstruction of ACL using TT technique [14–16].

In this study, we hypothesized that TT technique can be applied safely to anatomical reconstruction of ACL when the knee is secured at 90° of flexion and ACL spatial structure analogy could be simulated by double-sided laser technique to precisely depict the position of the tibial-femoral tunnel.

## Methods

### Subjects

This study was approved by The Affiliated Suzhou Hospital of Nanjing Medical University Ethics Committee. No written/verbal consent was needed for this study because cadavers were used. Forty-nine intact knees of 25 cadavers were used in this study because one knee was found to have slight osteoarthritis and was excluded. The subjects included 19 males and 6 females, mean age was 59.52 ± 11.13 years old, and mean height was 164.92 ± 7.27 cm.

### Dissection

The body was put in a supine position, the skin and the fat tissue were carefully removed, and the quadriceps femoris and patellar tendon were identified. The patellar tendon was cut after its length was measured and stripped along the patella, close to the femur and underneath the quadriceps femoris until the shaft of the femur was exposed. Next, synovial membranes and fat pads were cleaned carefully and ACL was exposed.

### Measurement

**Fig. 1:**
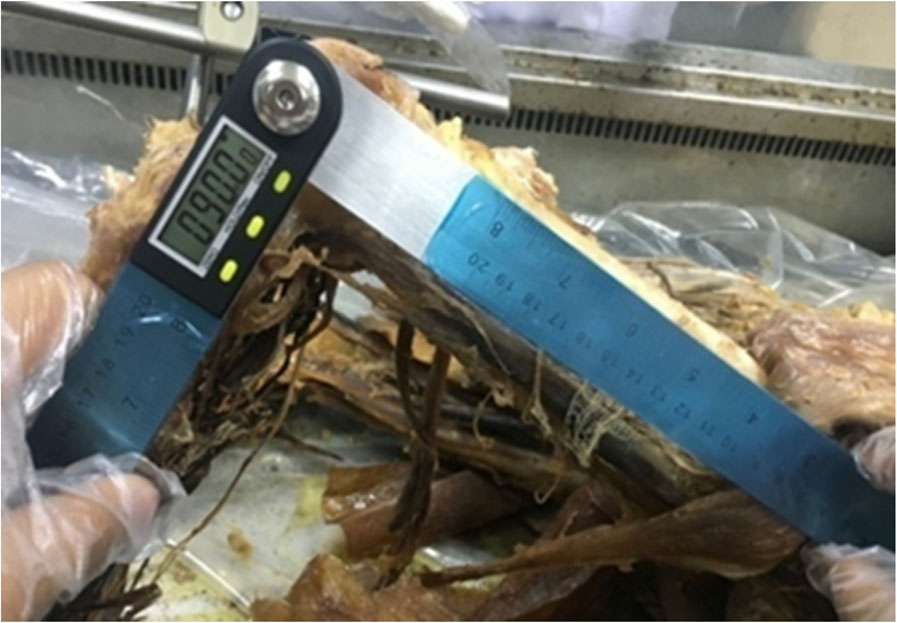
Measurement with the knee secured at 90°

**Fig. 2:**
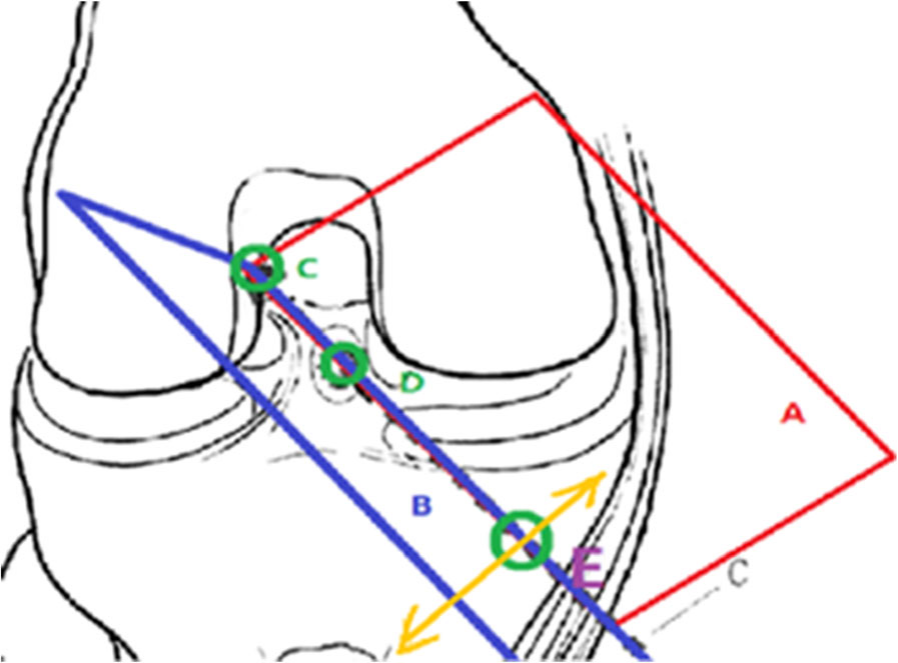
Double-sided laser technology. One of the ACL spatial configuration locating methods. The central point of ACL was located and marked with gentian violet. The point C and D was aligned to create plane A using high-accuracy surface-type laser transmitter; The point C and D was aligned to create plane B using another high-accuracy surface-type laser transmitter; Plane A and B intersected a spatial line L, and line L passed through point C and D, and point C and D defined the ACL spatial configuration. Line L passed through the tibial exit point and femoral exit point as E and F, respectively. If the tunnel is straight, then CDEF is on the same line

**Fig. 3:**
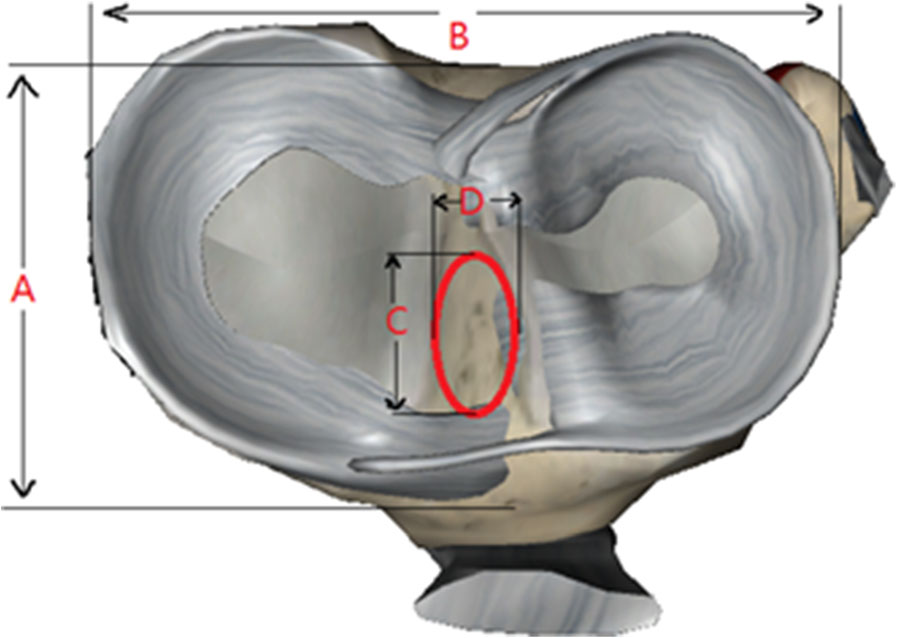
The measurement of the tibia (**a**). Transverse diameter (**b**). Anteroposterior diameter (**c**, **d**). The anteroposterior length and the maximum width of the right knee ACL tibial attachment, respectively

**Fig. 4:**
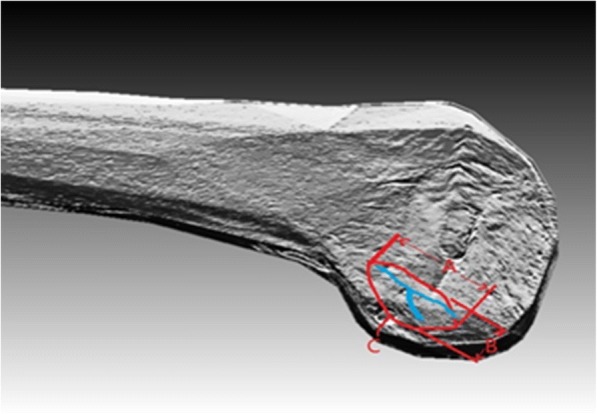
The measurement of the femur. **a** The length of the femoral attachment. **b** The width of the femoral attachment. **c** The distance from the femoral attachment to the posterior wall. The lateral intercondylar eminence and lateral furcatus eminence were indicated by blue

The knee flexion angle was measured with a digital goniometer (0–200 mm, ELECALL). One arm of the goniometer was aligned with the long axis of femur shaft, and the other arm of the goniometer was aligned with the long axis of tibia shaft. The measurement was taken at 90° of knee flexion (Fig. 1). No horizontal and lateral torques were applied. The position of tibia and femur was evaluated to avoid the rotation of tibia and femur. Single-side external fixation supporter was used to hold the internal and external of the knee joint securely and avoid the translocation of the knee joint. After fixation, ACL was removed carefully. The central point in footprints was chosen as the central point of the tunnel. Two high-accuracy laser transmitters (Yuan Ad LASER, 650 nm, type YD-L650P100-26-110) were used to create a laser plane. The central point of ACL was located and marked with gentian violet. The point C and D was aligned to create plane A using high-accuracy surface-type laser transmitter; The point C and D was aligned to create plane B using another high-accuracy surface-type laser transmitter; Plane A and B intersected a spatial line L, and line L passed through point C and D, and point C and D defined the ACL spatial configuration. Line L passed through tibial exit point and femoral exit point as E and F, respectively. If the tunnel is straight, then CDEF is on the same line (Fig. 2). The measurement of the tibia and femur was demonstrated in Figs. 3 and 4, respectively.

### Statistical analysis

Statistical analysis was performed using SPSS 21.0 (SPSS, Inc., Chicago, IL, USA). Data were presented as X ± SD. The continuous variables were tested by Kolmogorov–Smirnov to analyze the normality and by Levene’s test to analyze the homogeneity. Groups were compared by independent *T* test. *P* < 0.05 indicated significant difference.

## Results

In this study, we examined 49 knees from 25 subjects, the male to female ratio was 19:6, the mean age was 59.52 ± 11.13 years old, the mean height was 164.92 ± 7.27 cm, the length of the patellar tendon was 35.23 ± 5.10 mm, the tibial transverse diameter was 73.50 ± 4.89 mm, the tibial anteroposterior diameter was 45.18 ± 4.01 mm; the length of tibial attachment was 15.75 ± 2.44 mm; the width of tibial attachment was 7.80 ± 1.28 mm; and the distance from the femoral attachment to the posterior wall was 2.61 ± 0.62 mm. The occurrence of the lateral intercondylar eminence was 76%, and the occurrence of the lateral furcatus eminence was 49%.

For ACL tunnel reconstruction, at the 90° of flexion, Kirschner wires were drilled through point D and E into the central point of the femoral ACL footprint, then drilled out around point F. The mean distance to point F was 1.14 ± 0.82 mm, the length of the tibial tunnel was 31.83 ± 4.09 mm, the distance to the tibial plateau was 16.33 ± 4.56 mm, and the distance to the patellar tendon was 10.79 ± 5.85 mm; the distance to the medial collateral ligament was 23.12 ± 5.99 mm; and the length of the femoral tunnel was 42.70 ± 7.83 mm. The comparison of left knees and right knees showed no significant difference (Table 1).

**Table 1:** The comparison of left and right knee anatomical data

	Left knee	Right knee	*T*	*P*
Length of patellar tendon	35.28 ± 4.87	35.30 ± 5.49	− 0.01	0.99
Tibial anteroposterior diameter	45.33 ± 4.06	45.04 ± 4.13	0.24	0.81
Tibial transverse diameter	73.25 ± 5.14	73.71 ± 4.82	− 0.32	0.75
Length of tibial attachment	15.74 ± 2.31	15.95 ± 2.48	− 0.31	0.76
Width of tibial attachment	7.96 ± 1.25	7.98 ± 1.35	− 0.06	0.96
Length of femoral attachment	15.33 ± 2.15	15.34 ± 2.21	0.01	0.99
Width of femoral attachment	8.97 ± 1.74	8.89 ± 1.50	0.18	0.86
Distance from the femoral attachment to the posterior wall	2.61 ± 0.68	2.61 ± 0.59	0.04	0.97
Distance from laser point to Kirschner wire point	1.06 ± 0.85	1.21 ± 0.81	− 0.60	0.55
Length of tibial tunnel at 90°	31.94 ± 4.26	31.47 ± 3.88	0.39	0.47
Distance from point E to joint line at 90°	16.01 ± 4.12	16.92 ± 4.92	− 0.69	0.90
Distance from point E to patellar tendon at 90°	10.92 ± 5.57	10.81 ± 6.30	0.06	0.11
Distance from point E to medial collateral ligament at 90°	23.63 ± 5.74	22.63 ± 6.42	0.57	0.57
Length of femoral tunnel at 90°	42.54 ± 7.86	42.33 ± 7.88	0.09	0.92

The unit was millimeter

## Discussion

According to the anatomical reconstruction, the bundle could be classified to single bundle, double bundles, and triple bundles. In this study, we used single-bundle because we found that the length of ACL tibial attachment was 15.75 ± 2.44 mm and the length of femoral attachment was 15.40 ± 2.17 mm, which were not the indication for the use of double bundles. Previous studies suggested that the double bundles can be safely conducted if the long axis of the anatomical footprint is greater than 16 mm. However, if the width of ACL footprint is less than 14 mm, the double bundle cannot be conducted [17, 18]. In addition, the double-bundle technique cannot be applied to severe open bone contusion, notch structure, severe arthritis, or multiple injuries, and the surgery is complicated [19].

A meta-analysis of 22 studies compared the difference between single- and double-bundle anatomical reconstruction and found that the double-bundle anatomical reconstruction only had the advantage of rotational stability, and most clinical function outcomes except IKDC score showed no significant difference between single- and double-bundle ACL reconstruction [7]. Therefore, in this study, we chose the single-bundle ACL anatomical reconstruction.

Recent studies showed that the thicker graft has a lower failure rate for ACL reconstruction [20–22]. However, the width of the grafts should be limited. A recent study suggested that the size of the tunnel was determined by the size of ACL footprint. For example, if the length of the tibial attachment in implant position was 18 mm and the width was 8 mm, 8-mm width was recommended as the diameter of the tunnel [23]. In this study, the width of the tibial attachment was 7.80 ± 1.28 mm and the width of the femoral attachment was 8.97 ± 1.61 mm in the subjects. Thus, the width for ACL single-bundle reconstruction should be around 7.8 mm for people in our region.

In this study, we chose tibial tunnel technique to conduct anatomical reconstruction. This technology is simple and safe, has decreased risk of revision compared to anteromedial technique, and has been widely applied [24, 25]. However, some researchers doubted the possibility of transtibial tunnel technique to achieve anatomical reconstruction of ACL [26, 27]. Several studies showed that revised transtibial tunnel technique could achieve anatomical reconstruction of ACL [28, 29]. In this study, we successfully used transtibial tunnel technique to achieve anatomical reconstruction of ACL.

The occurrence of the lateral intercondylar eminence was 76% and that of the lateral furcatus eminence was 49%; these anatomical markers are permanent and could be used as the markers of ACL anatomical reconstruction. We used double-sided laser technology in our measurement, which is simple, accurate, and cheap and can evaluate the tunnel from different angles. The distance from Kirschner wires’ exit position to the lateral point of the femur was only 1.14 ± 0.82 mm.

This study has several limitations. First, the age of the subjects is biased and all subjects were middle-aged or elders. Second, the sample size is limited. Third, we could not exclude some confounding factors that affect the measurement of ACL. Further large-scale studies are needed to prove the application of double-sided laser technology and transtibial technique to single-bundle anatomical reconstruction of ACL.

## Conclusions

In summary, our study suggests that for subjects in the southern region of Jiangsu, China, transtibial tunnel technique can be used to achieve single-bundle ACL anatomical reconstruction. Because tibial tunnel restrains the direction and the angle of the femoral tunnel, great care should be taken during the reconstruction. We recommend the use of new type of ACL locator with laser positioning during drilling to decrease the failure rate. Lateral intercondylar eminence can be used as the anatomical marker during the reconstruction.

## Abbreviations

ACL: Anterior cruciate ligament
OI: Outside-in
TP: Trans-portal
TT: Transtibial tunnel

## References

[1] Nordenvall R, Bahmanyar S, Adami J, Stenros C, Wredmark T, Felländer-Tsai L (2012). A population-based nationwide study of cruciate ligament injury in Sweden, 2001-2009: incidence, treatment, and sex differences. Am J Sports Med.

[2] Ajuied A, Wong F, Smith C (2014). Anterior cruciate ligament injury and radiologic progression of knee osteoarthritis: a systematic review and meta-analysis. Am J Sports Med.

[3] Zavras TD, Race A, Bull AM, Amis AA (2001). A comparative study of ‘isometric’ points for anterior cruciate ligament graft attachment. Knee Surg Sports Traumatol Arthrosc.

[4] Riboh JC, Hasselblad V, Godin JA, Mather RC (2013). Transtibial versus independent drilling techniques for anterior cruciate ligament reconstruction: a systematic review, meta-analysis, and meta-regression. Am J Sports Med.

[5] Whitehead TS (2013). Failure of anterior cruciate ligament reconstruction. Clin Sports Med.

[6] Crawford SN, Waterman BR, Lubowitz JH (2013). Long-term failure of anterior cruciate ligament reconstruction. Arthroscopy.

[7] Zhang Y, Xu C, Dong S, Shen P, Su W, Zhao J (2016). Systemic review of anatomic single- versus double-bundle anterior cruciate ligament reconstruction: does femoral tunnel drilling technique matter?. Arthroscopy.

[8] Kilinc BE, Kara A, Oc Y (2016). Transtibial vs anatomical single bundle technique for anterior cruciate ligament reconstruction: a retrospective cohort study. Int J Surg.

[9] Rahr-Wagner L, Thillemann TM, Pedersen AB, Lind MC (2013). Increased risk of revision after anteromedial compared with transtibial drilling of the femoral tunnel during primary anterior cruciate ligament reconstruction: results from the Danish Knee Ligament Reconstruction Register. Arthroscopy.

[10] Clockaerts S, Van Haver A, Verhaegen J (2016). Transportal femoral drilling creates more horizontal ACL graft orientation compared to transtibial drilling: a 3D CT imaging study. Knee.

[11] Park JS, Park JH, Wang JH (2015). Comparison of femoral tunnel geometry, using in vivo 3-dimensional computed tomography, during transportal and outside-in single-bundle anterior cruciate ligament reconstruction techniques. Arthroscopy.

[12] Robin BN, Jani SS, Marvil SC, Reid JB, Schillhammer CK, Lubowitz JH (2015). Advantages and disadvantages of transtibial, anteromedial portal, and outside-in femoral tunnel drilling in single-bundle anterior cruciate ligament reconstruction: a systematic review. Arthroscopy.

[13] Luzo MV, Franciozi CE, Rezende FC, Gracitelli GC, Debieux P, Cohen M (2016). Anterior cruciate ligament—updating article. Rev Bras Ortop.

[14] Usman MA, Kamei G, Adachi N, Deie M, Nakamae A, Ochi M (2015). Revision single-bundle anterior cruciate ligament reconstruction with over-the-top route procedure. Orthop Traumatol Surg Res.

[15] Kopf S, Forsythe B, Wong AK, Tashman S, Irrgang JJ, Fu FH (2012). Transtibial ACL reconstruction technique fails to position drill tunnels anatomically in vivo 3D CT study. Knee Surg Sports Traumatol Arthrosc.

[16] Bhatia S, Korth K, Van Thiel GS (2016). Effect of tibial tunnel diameter on femoral tunnel placement in transtibial single bundle ACL reconstruction. Knee Surg Sports Traumatol Arthrosc.

[17] Pombo MW, Shen W, Fu FH (2008). Anatomic double-bundle anterior cruciate ligament reconstruction: where are we today?. Arthroscopy.

[18] Siebold R (2011). The concept of complete footprint restoration with guidelines for single- and double-bundle ACL reconstruction. Knee Surg Sports Traumatol Arthrosc.

[19] Snow M, Stanish WD (2010). Double-bundle ACL reconstruction: how big is the learning curve?. Knee Surg Sports Traumatol Arthrosc.

[20] Dai C, Wang F, Wang X, Wang R, Wang S, Tang S (2016). Arthroscopic single-bundle anterior cruciate ligament reconstruction with six-strand hamstring tendon allograft versus bone-patellar tendon-bone allograft. Knee Surg Sports Traumatol Arthrosc.

[21] Conte EJ, Hyatt AE, Gatt CJ, Dhawan A (2014). Hamstring autograft size can be predicted and is a potential risk factor for anterior cruciate ligament reconstruction failure. Arthroscopy.

[22] Magnussen RA, Lawrence JT, West RL, Toth AP, Taylor DC, Garrett WE (2012). Graft size and patient age are predictors of early revision after anterior cruciate ligament reconstruction with hamstring autograft. Arthroscopy.

[23] Martins CAQ, Kropf EJ, Shen W, van Eck CF, Fu FH (2008). The concept of anatomic anterior cruciate ligament reconstruction. Oper Tech Sports Med.

[24] Lee S, Kim H, Jang J, Seong SC, Lee MC (2012). Comparison of anterior and rotatory laxity using navigation between single- and double-bundle ACL reconstruction: prospective randomized trial. Knee Surg Sports Traumatol Arthrosc.

[25] Fujita N, Kuroda R, Matsumoto T (2011). Comparison of the clinical outcome of double-bundle, anteromedial single-bundle, and posterolateral single-bundle anterior cruciate ligament reconstruction using hamstring tendon graft with minimum 2-year follow-up. Arthroscopy.

[26] Chang CB, Choi JY, Koh IJ, Lee KJ, Lee KH, Kim TK (2011). Comparisons of femoral tunnel position and length in anterior cruciate ligament reconstruction: modified transtibial versus anteromedial portal techniques. Arthroscopy.

[27] Kopf S, Forsythe B, Wong AK (2010). Nonanatomic tunnel position in traditional transtibial single-bundle anterior cruciate ligament reconstruction evaluated by three-dimensional computed tomography. J Bone Joint Surg Am.

[28] Lee SR, Jang HW, Lee DW, Nam SW, Ha JK, Kim JG (2013). Evaluation of femoral tunnel positioning using 3-dimensional computed tomography and radiographs after single bundle anterior cruciate ligament reconstruction with modified transtibial technique. Clin Orthop Surg.

[29] Youm YS, Cho SD, Lee SH, Youn CH (2014). Modified transtibial versus anteromedial portal technique in anatomic single-bundle anterior cruciate ligament reconstruction: comparison of femoral tunnel position and clinical results. Am J Sports Med.

